# Vehicle-to-vehicle cooperative driving model considering end-to-end delay of communication network

**DOI:** 10.1038/s41598-023-49365-x

**Published:** 2023-12-27

**Authors:** Yi-rong Kang, Yijun Chen, Chuan Tian

**Affiliations:** 1 School of Transportation Engineering, Guizhou Institute of Technology, Guiyang, 550003 China; 2 School of Economics and Finance, Guizhou University of Commerce, Guiyang, 550014 China

**Keywords:** Engineering, Mathematics and computing, Physics

## Abstract

To explore the mechanism of the end-to-end transmission delay of the communication network on the collaborative driving process for traffic flow in the vehicle-to-vehicle communication environment, based on the idea of the car-following model, this paper introduces characteristic parameters characterizing the end-to-end transmission delay of the network into Newell's following model and proposes a CD and OV model by considering the time delay characteristics of the collaborative driving process from information transmission to control decision and then to physical execution. To determine the cooperative driving system's stability criterion, the stability analysis of the new model is examined. By using the reductive perturbation approach, the spatiotemporal evolution mechanism of the traffic flow around the critical stability point under the influence of various transmission delays is analyzed. The resulting modified Korteweg-de Vries (mKdV) equations and density wave solutions are derived. The results show that the end-to-end transmission delay of the network has a significant shock effect on the stability of the vehicle-vehicle cooperative driving system, and the stability of the traffic flow and the ability to suppress traffic congestion becomes worse with the increase in the end-to-end transmission delay.

## Introduction

The traffic flow model has become an important tool to quantitatively study the formation and evolution mechanisms of traffic congestion. According to the modeling scale of variables, traffic flow models can be divided into micro traffic flow models and macro traffic flow models. The micro traffic flow models mainly include the car following model^1,2^ and the cellular automata model^3,4^. The macro traffic flow model mainly includes the continuum model^5,6^ and lattice model^7,8^. The car-following model mainly studies the interaction mechanisms of preceding and following vehicles in a single lane. The general car following model uses simple differential equations to describe the motion law of a single vehicle in the following process. By analyzing differential equations or numerical simulation, it can accurately capture the spatio-temporal motion characteristics of vehicles. Therefore, it has attracted the extensive attention of scholars.

After decades of development, the car following model has achieved fruitful research results. Among them, Newell^2^ constructed a stimulus–response logic framework to simulate the motion trajectories in the process of car following. In 1995, Bando^9^ creatively established the optimal velocity (OV) model by introducing the concept of optimized speed function. The OV model successfully portrayed the interaction process between the current vehicle and its preceding car on a single lane. In 1998, Helbing and Tilch^10^ conducted a fitting study of the OV model using field data and found unrealistic deceleration and vehicle collision behavior in the OV model. To overcome the defects, they established the generalized force (GF) model based on the OV model by introducing the negative speed difference effect between the front and rear consecutive vehicles. Thereafter, Rui^11^ further extended the GF model by introducing the positive speed difference factor and proposing the famous full velocity difference (FVD) model. Since then, the OV model and the FVD model have become the two basic models most widely used in the car following theory. For example, Zhai et al.^12^ proposed an extension of the OV model with explicit consideration of the driver’s characteristics (timid and aggressive driving behavior) and the traffic jerk effect. Further, based on the continuum model, Zhai et al.^13^ conducted a systematic study of the effect of drivers' characteristics and traffic jerks on the traffic flow at the macroscale. Peng et al.^14^ developed a new optimal velocity difference (OVD) model for a car-following theory based on the FVD model. The research results of the OVD model indicate that it has successfully overcome the negative velocity defect that appears in the FVD model. By introducing acceleration difference terms into the FVD model, Zhao et al.^15^ presented a full velocity and acceleration difference (FVAD) model. The main improvement over the previous models is that the FVAD model can exactly describe the driver’s behavior in an urgent case where no collision occurs and no unrealistic deceleration appears in this model. Li et al.^16^ analyzed a new car-following model by incorporating the opening angle of the electronic throttle into the FVD model. Literature^17,18^ investigated the "backward-looking" properties of an extended OV model. Zhai et al.^19^ proposed a new car-following model, taking the predictive headway variation and preceding vehicle’s taillight effect into the FVD model. On this basis, the corresponding continuous traffic flow model is constructed. It is well known that road morphology has a great influence on vehicle dynamics and traffic flow performance. For this reason, some scholars have improved the car-following model by introducing factors related to the road environment. Zhai et al.^20^ developed a modified car-following model considering perceived headway errors on gyroidal roads based on the OV model, and an effective controller was designed to reduce traffic congestion using electronic throttle information as the stability condition is unsatisfied. Zhu et al.^21^ investigated the slope effects upon traffic flow on a single-lane gradient (uphill or downhill) highway, and the stability and density waves of the traffic flow on a gradient highway with different slopes were obtained. Recently, starting from the FVD model, Zhai et al.^22^ built a new traffic flow model accounting for the uncertainty of the preceding vehicle’s velocity on gradient roads and then designed a new self-delayed feedback controller by using the velocity and headway differences between the current time step and the historical time step. Sun et al. proposed an extended car-following model by taking the effect of electronic throttle dynamics into account on the curved road, in which the electronic throttle opening angle difference from multiple preceding vehicles at the previous moment is considered as a delay-feedback control signal. In addition, based on the car following theory, some scholars have begun to study the control methods of emissions and energy consumption during the vehicle driving process. Zhai et al.^24^ designed a new periodic intermittent cruise controller to track and assess the real-time operating status and relevant environmental indicators of road vehicles, in which the framework of the FVD model is adopted to depict the motion process of vehicles on the road. The results show that the newly designed controller is effective in reducing exhaust emissions and energy consumption, as well as in improving vehicle safety tracking performance. Based on the coupled map car-following model, Zhang et al.^25^ presented a discrete version of the car-following model by introducing a relative speed term and then designed a delay-feedback controller to suppress traffic jams and decrease traffic emissions.

With the rapid development of communication technology, the transportation system is moving towards the age of vehicle-to-vehicle cooperative driving with real-time information exchange between vehicles. Under this new context, the car following traffic flow theory will undergo major changes. Subsequently, a series of extended car following models describing the behavior of the vehicle-to-vehicle (V2V) environment have been proposed successively. Lenz et al.^26^ and Nakayama et al.^27^ extended the OV model with forward-looking and backward-looking effects in a cooperative driving system. Yu et al.^28^ proposed multi-anticipative car following models by incorporating multi-vehicle interactions. Sun et al.^29^ verified that the stability of traffic flow could be enhanced when the average speed of several preceding cars was considered. Tang et al.^30^ proposed a car-following model under the inter-vehicle communication (IVC) system. Zhang et al.^31^ presented a CV (Internet-Connected Vehicle) platoon car-following model that has a synthesized optimal velocity differential function and a full platoon with a weighted relative velocity difference function. Zhu et al.^32^ investigated the mixed traffic flow with human-driving and autonomous cars based on the car-following model. The fundamental diagrams and density waves of the mixed traffic flow were investigated. There is also literature that investigates additional characteristics of the vehicle-to-vehicle cooperative driving car-following models^33–35^. These research results have laid a foundation for people to simulate and recognize the process of vehicle cooperative driving in a V2V environment from different aspects.

However, it should be pointed out that the above-mentioned car following models built for V2V environments are established under the ideal communication environment, which is inconsistent with the actual characteristics of the communication environment operation. Recently, a few scholars have begun to pay attention to the influence of factors related to real vehicular networks on car-following motion. For instance, to address the cyber-attack factor in the connected vehicle environment, Zhai et al.^36^ designed a continuous delay feedback controller based on the lattice model to guarantee the stability of the traffic flow operation. In this paper, we focus on the influence of the end-to-end transmission delay of the communication network (the time required for the signal to be sent from the source node to be received by the target node) on the physical motion process of the vehicle in real-world traffic scenarios. So far, to the best of our knowledge, this intrinsic factor of the transmission network has not been studied in existing traffic flow models. The transmission delay of a communication network is an inevitable and non-negligible objective factor^35,37^, and its time delay effect will inevitably be mapped to the physical movement process of vehicles, which will have an oscillatory impact on the stability and control quality of traffic flow and reduce the performance of the vehicle-to-vehicle cooperative driving system. At the same time, research in the control field shows that^38^, the transmission delay of the communication network has a great impact on the performance of the control system, which may lead to the instability and collapse of the control system. However, there is a lack of literature to explore the influence of end-to-end transmission delay in the communication network on the characteristics of the vehicle-to-vehicle cooperative driving process in a V2V environment. Therefore, this paper draws lessons from the basic idea of the car following model and, based on the classical Newell car following model^2^, considers the time-delay characteristics of end-to-end transmission delay of the network on the movement process of vehicles, introduces the characteristic parameters of network delay, and constructs a new vehicle cooperative driving model. On this basis, theoretical analysis and numerical simulation are used to study the effect of end-to-end transmission delay on the collaborative driving process for traffic flow in a V2V environment.

## Models

### Newell car following model

In 1961, Newell^2^ proposed the vehicle motion equation for the single-lane car with the following system:

1 $$v_{j} (t + \tau ) = V(\Delta x_{j} (t))$$

where, $\tau$ is the delay time, including the reaction delay of the driver and the mechanical delay of vehicle adjustment,$t$ represents the time variable and the subscript $j$ is the vehicle label. $x_{j} (t)$ and $x_{j + 1} (t)$ are the position variables of the following vehicle and the preceding vehicle respectively. $v_{j} (t)$ represents the speed of the jth vehicle. $\Delta x_{j} = x_{j + 1} (t) - x_{j} (t)$ is the headway between the preceding vehicle $j + 1$ and the following vehicle $j$ at the time $t$. $V(\Delta x_{j} )$ represents the optimal speed function. The basic idea of the Newell model is that the speed $v_{j} (t + \tau )$ of the vehicle at the time $t + \tau$ is determined by the optimal speed $V(\Delta x_{j} (t))$ that can be achieved by the headway $\Delta x_{{\text{j}}}$ at the time $t$, which is adjusted by the driver within the delay time $\tau$. The significance of the Newell model is that it puts forward the idea that there is an optimal speed depending on the headway in the process of car following, which provides a new framework for subsequent car following model modeling.

The differential equation consistent with the OV model put forth by Bando^9^ can be obtained by first-order Taylor expansion of Eq. (1) of the Newell model^2^.

2 $$\frac{{dv_{j} (t)}}{dt} = a\left[ {V(\Delta x_{j} (t)) - v_{j} (t)} \right]$$

where, $a = 1/\tau$ is the sensitivity coefficient.

### The new model

With the rapid development of wireless communication, control, and artificial intelligence technology, cooperative driving technology in a V2V environment has become an important way to effectively improve the operation quality of traffic flow. Based on the V2V communication system, drivers can realize information interaction and real-time perception of the traffic situation ahead and adjust their driving behavior accordingly. Compared with traditional single-vehicle independent driving, vehicle-to-vehicle cooperative driving has strict transmission delay requirements for the communication system. The research results show that the end-to-end delay required by traditional safety-related services is less than 100 ms, but in the scenario of a vehicle-to-vehicle cooperative driving system, the end-to-end delay of V2V is less than 1 ms^39^. It can be seen that the end-to-end transmission delay of the communication system is a sensitive factor in the process of cooperative driving and a key variable to be considered in constructing the cooperative driving model in a V2V environment.

Based on the V2V platform, after the sensor collects the status information at time *t*, it must experience a network transmission delay $\psi$ before it can be received by the driver /controller. After the driver/controller experiences the reaction delay and the vehicle mechanical delay (the total delay of these two parts is $\tau$), the response or control decision to the status information at time *t* can act on the physical process of the vehicle and lead to a change in the physical state of the vehicle. Therefore, the process of vehicle-to-vehicle cooperative driving in a V2V environment has experienced a time delay from information sensing to control decisions and then to physical execution. From the above analysis process, it can be seen that the information measured at time *t* will affect the physical state of the vehicle at the time $t + \tau + \psi$. Therefore, based on the idea of the car following theory and the above analysis, we introduce the end-to-end transmission delay factor of the transmission network into the framework of the Newell model^2^ and propose a new model called CD and OV (communication delay and optimal velocity model). Its motion equation is as follows:

3 $$v_{j} (t + \tau + \psi ) = V(\Delta x_{j} (t))$$

For the convenience of analysis, we set the relation $\psi = \delta \tau$ between transmission delay $\psi$ and the variable $\tau$. By Taylor expansion of the variables $v_{n} (t + \tau + \psi )$ on the left of Eq. (3) at point $t$ and ignoring the nonlinear terms, the following formula is obtained:

4 $$v_{j} (t + \tau + \psi ){ = }v_{j} [t + (1 + \delta )\tau ] = v_{j} (t) + (1 + \delta )\tau \frac{{dv_{j} (t)}}{dt}$$

Substituting Eq. (4) into Eq. (3), the following expression is obtained:

5 $$\frac{{dv_{j} (t)}}{dt} = \frac{a}{1 + \delta }\left[ {V(\Delta x_{j} (t)) - v_{j} (t)} \right]$$

where $a = 1/\tau$($\tau > {0}$)is the sensitivity coefficient, the range of the parameter $\delta$ is a non-negative real number, and its value depends on the specific vehicle network environment. As $\delta = 0$, the CD and OV model degenerated into the OV model.

For subsequent analysis, we rewrite Eq. (5) into the form of headway.

6 $$\frac{{d^{2} \Delta x_{j} (t)}}{{dt^{2} }} = \frac{a}{1 + \delta }[V(\Delta x_{j + 1} (t)) - V(\Delta x_{j} (t)) - \frac{{d\Delta x_{j} (t)}}{dt}]$$

In this study, the optimal velocity function $V(\Delta x_{j} )$ is adopted as follows^22^:

7 $$V(\Delta x) = V_{1} + V_{2} \tanh \left[ {C_{1} (\Delta x - l_{c} ) - C_{2} } \right]$$

where $l_{c}$ is the vehicle length, $l_{c} = 5m$ in the simulation. Other parameters are set as $V_{1} = 6.75m/s$, $V_{2} = 7.91m/s$, $C_{1} = 0.13m^{ - 1}$, $C_{2} = 1.57$.

In the new model, it is crucial to distinguish between the end-to-end transmission delay of the communication network, the driver's reaction delay, and the mechanical delay of vehicle adjustment. The key distinctions among them are as follows:

(a) End-to-end transmission delay mainly refers to the total time required from the generation of information at the source in the vehicular network to the complete reception of the information at the destination. It usually includes delays in multiple stages, such as sending, propagation, processing, and queuing. (b) The driver's reaction delay refers to the time required from the time a driver receives a stimulus (e.g., a warning or other vehicle maneuver) to the time he or she responds (e.g., by turning the steering wheel or applying the brakes). This aspect is commonly associated with human perception, cognition, and decision-making processes. (c) The mechanical delay of vehicle adjustment refers to the fact that when the driver makes some action (e.g., applying the brakes), it takes some time for the vehicle to complete this action (e.g., coming to a complete stop). This depends on the physical nature of the vehicle, such as weight, power, and mechanical design.

## Stability analysis

In order to explore the influence of end-to-end transmission delay on the stability of the traffic flow system, the stability analysis is carried out below. At the initial time, it is assumed that all vehicles move on a circular road at a uniform speed with headway $b$ and optimal speed $V(b)$. Obviously, at this time, the traffic flow corresponding to the CD and OV model is in an equilibrium state, and the coordinates of its steady-state solution can be expressed as follows:

8 $$x_{j}^{0} (t) = bj + V(b)t,\,\,b = L/N$$

where $N$ represents the total number of vehicles on the road, and the $L$ is the length of the ring road. To study the stability of the traffic flow under the small disturbance condition, the small disturbance signal $y_{j} (t)$ is applied to make the traffic flow produce motion deviation.

9 $$x_{j} (t) = x_{j}^{0} (t) + y_{j} (t)$$

Bring Eq. (9) into Eq. (6), and then linearize it to give that

10 $$y^{\prime\prime}_{j} (t) = \frac{a}{1 + \delta }[V^{\prime}(b)\Delta y_{j} (t) - y^{\prime}_{j} (t)]$$

where $V^{\prime}(b) = dV(\Delta x_{j} )/d\Delta x_{j} \left| {_{{\Delta x_{j} = b}} } \right.$, $\Delta y_{j} (t) = y_{j + 1} (t) - y_{j} (t)$. For simplicity, $V^{\prime}(b)$ are abbreviated as $V^{\prime}$.

Expand the function $y_{j}$ into a Fourier series and take its monochromatic component: $\Delta y_{j} (t) = Ae^{ikj + zt}$, expand the parameters $z$ in the form of $z = z_{1} (ik) + z_{2} (ik)^{2} + \cdots$ at the same time, and obtain the first-order and second-order terms as follows,

11 $$z_{1} = V^{\prime}(b), \, z_{2} = \frac{{V^{\prime}(b)}}{2} - \frac{{(1 + \delta )z_{1}^{2} }}{a}$$

According to the long wave theory, if $z_{2}$ is negative, the initial uniformly stable traffic flow will be unstable under small disturbances. On the contrary, the original equilibrium state will remain unchanged. Therefore, as $z_{2} = 0$, the critical stability conditions of the CD and OV model can be obtained as follows:

12 $$a = 2(1 + \delta )V^{\prime}(b)$$

When the headway meets the following relationship, the system will be in a stable state:

13 $$a > 2(1 + \delta )V^{\prime}(b)$$

When $\delta = 0$, the stability criterion consistent with the OV model^9^ was obtained.

**Figure 1:**
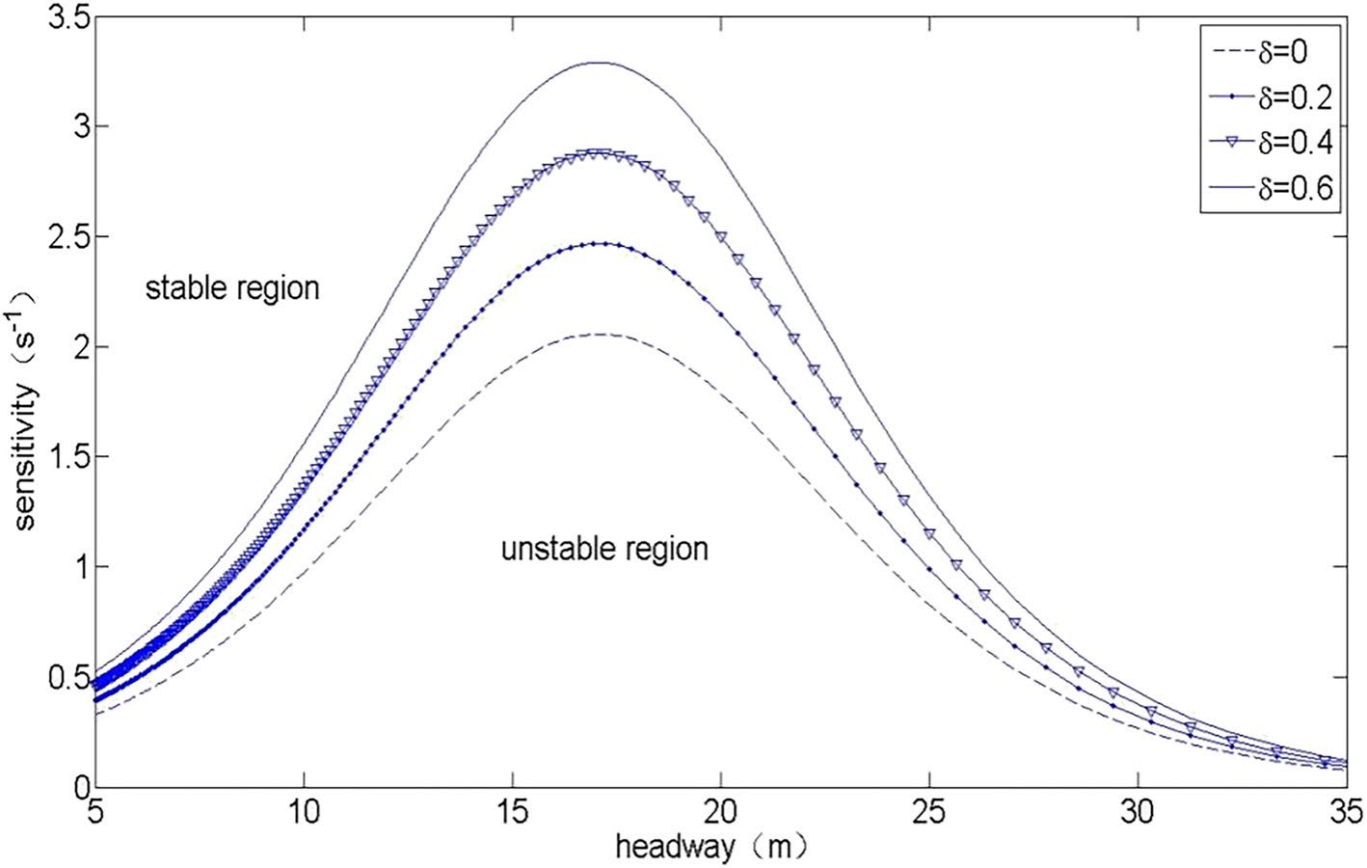
The neutral stability curves for the CD and OV model in headway-sensitivity space with different $\delta$.

Figure 1 shows the neutral stability curve of the CD and OV model under different network end-to-end transmission delays $\delta$. It can be seen from Fig. 1 that the neutral stability curve divides the headway-sensitivity space into two parts: the upper part corresponds to the stable region, and the lower part corresponds to the unstable region. In the stable region, the small disturbance signal will gradually decline with the development of time in the process of the car following and finally return to a steady state. In the unstable area, the small disturbance signal will propagate along the upstream and gradually amplify in the process of traffic flow movement, finally forming a traffic jam. It can be seen from Fig. 1 that the unstable area gradually amplifies with the increase in network delay $\delta$, which indicates that the stability of the vehicle-to-vehicle cooperation driving process gradually deteriorates with the increase in V2V transmission network delay. In particular, as $\delta = 0$, the neutral stability curve is consistent with that in the OV model^9^.

## Nonlinear stability analysis

In order to reveal the influence of the network transmission delay effect on the temporal and spatial evolution characteristics of the area near the critical stability curve $(h_{c} ,a_{c} )$, the nonlinear analysis method is adopted in the following investigation. Therefore, near the critical stability curve $(h_{c} ,a_{c} )$, for space $j$ and time t, two slow variables $X$ and $T$ are introduced, which are defined as follows:

14 $$X = \varepsilon (j + bt),\,\,T = \varepsilon^{3} t,\,\,\,0 < \varepsilon \le 1$$

where $b$ is a constant to be determined and $\varepsilon$($0 < \varepsilon \ll 1$) is a small positive scaling parameter. Assuming that the variable satisfies the following equation,

15 $$\Delta x_{j} (t) = h_{c} + \varepsilon R(X,T)$$

By substituting formulas (14) and (15) into Eq. (6) and then making Taylor expansion to the fifth order of $\varepsilon$ for each term, one can obtain:

16 $$\begin{gathered} \varepsilon^{2} (b - V^{\prime})\partial_{X} R + \varepsilon^{3} \left[ {\frac{{(1 + \delta )b^{2} }}{a} - \frac{{V^{\prime}}}{2}} \right]\partial_{X}^{2} R + \varepsilon^{4} \left[ {\partial_{T} R - \frac{{V^{\prime}}}{6}\partial_{X}^{3} R - \frac{1}{6}V^{\prime\prime\prime}\partial_{X} R^{3} } \right] \hfill \\ + \varepsilon^{5} \left[ {\frac{2(1 + \delta )b}{a}\partial_{T} \partial_{x} R - \frac{{V^{\prime}}}{24}\partial_{X}^{4} R - \frac{1}{12}V^{\prime\prime\prime}\partial_{X}^{2} R^{3} } \right] = 0 \hfill \\ \end{gathered}$$

where $V^{\prime} = [{{dV(\Delta x_{j} )} \mathord{\left/ {\vphantom {{dV(\Delta x_{j} )} {d\Delta x_{j} }}} \right. \kern-0pt} {d\Delta x_{j} }}]|_{{\Delta x_{j} = h_{c} }}$ and $V^{\prime\prime\prime} = [{{d^{3} V(\Delta x_{j} )} \mathord{\left/ {\vphantom {{d^{3} V(\Delta x_{j} )} {d\Delta x_{j}^{3} }}} \right. \kern-0pt} {d\Delta x_{j}^{3} }}]|_{{\Delta x_{j} = h_{c} }}$. Near the critical point $(\rho_{c} ,a_{c} )$, $\tau = (1 + \varepsilon^{2} )\tau_{c}$, taking $b = V^{\prime}$, the second-order and third-order terms in Eq. (16) can be eliminated to obtain the following simplified equation:

17 $$\varepsilon^{4} [\partial_{T} R - g_{1} \partial_{X}^{3} R + g_{2} \partial_{X} R^{3} ] + \varepsilon^{5} [g_{3} \partial_{X}^{2} R + g_{4} \partial_{X}^{4} R + g_{5} \partial_{X}^{2} R^{3} ] = 0$$

where

18 $$g_{1} = \frac{{V^{\prime}}}{6}$$

19 $$g_{2} = - \frac{{V^{\prime\prime\prime}}}{6}$$

20 $$g_{3} = (1 + \delta )b^{2} \tau_{c}$$

21 $$g_{4} = \frac{{(1 + \delta )bV^{\prime}\tau_{c} }}{3} - \frac{{V^{\prime}}}{24}$$

22 $$g_{5} = \frac{1}{12}[4(1 + \delta )b\tau_{c} - 1]V^{\prime\prime\prime}$$

In order to derive the standard mKdV equation, formula (17) is reformulated as follows:

23 $$T^{\prime} = g_{1} T, \, R = \sqrt {\frac{{g_{1} }}{{g_{2} }}} R^{\prime}$$

Therefore, we obtain the partial differential equation with higher-order infinitesimal terms $O(\varepsilon )$ as follows:

24 $$\partial_{{T^{\prime}}} R^{\prime} - \partial_{X}^{3} R^{\prime} + \partial_{X} R^{{\prime}{3}} + \varepsilon M[R^{\prime}] = 0$$

where

25 $$M[R^{\prime}] = \sqrt {\frac{1}{{g_{1} }}} [g_{3} \partial_{X}^{2} R^{\prime} + g_{4} \partial_{X}^{4} R^{\prime} + \frac{{g_{1} g_{5} }}{{g_{2} }}\partial_{X}^{2} R^{{\prime}{3}} ]$$

Ignoring the term $O(\varepsilon )$ in Eq. (25), we obtain the standard mKdV equation with the kink–antikink wave solution.

26 $$R^{\prime}_{0} (X,T^{\prime}) = \sqrt c \tanh \sqrt{\frac{c}{2}} (X - cT^{\prime})$$

where *c* denotes the propagation velocity. According to the method in reference^40^, the propagation velocity of a traffic jam wave can be obtained by integration:

27 $$c = \frac{{5g_{2} g_{3} }}{{2g_{2} g_{4} - 3g_{1} g_{5} }}$$

Therefore, the solution of the kink–antikink density wave of the CD and OV model is:

28 $$\Delta x_{j} (t) = h_{c} + \sqrt {\frac{{g_{1} c}}{{g_{2} }}\left( {\frac{\tau }{{\tau_{c} }} - 1} \right)} \tanh \sqrt {\frac{c}{2}\left( {\frac{\tau }{{\tau_{c} }} - 1} \right)} \left[ {j + (1 - cg_{1} \left( {\frac{\tau }{{\tau_{c} }} - 1} \right))t} \right]$$

The amplitude *A* of the kink–antikink wave solution is:

29 $$A = \sqrt {\frac{{g_{1} c}}{{g_{2} }}(\frac{\tau }{{\tau_{c} }} - 1)}$$

The nonlinear analysis results show that the cooperation driving traffic flow will be blocked by a small disturbance near the critical point $(h_{c} ,a_{c} )$ with considering the effect of end-to-end network transmission delay in a V2V environment. And the propagation speed and fluctuation amplitude of congestion are closely related to the transmission delay time $\delta$. The propagation law of traffic congestion can be described by the kink–antikink wave solution of the mKdV equation. As $\delta = 0$, the analytical result is consistent with that of the OV model^9^. The kink-antikink wave solution describes the nonlinear dynamic behavior and transmission laws of traffic congestion formation and dissipation. The kink wave represents a high-density, low-speed state of traffic, that is, traffic congestion. The antikink wave represents a low-density, high-speed state of traffic, that is, traffic fluidity. Thus, the kink-antikink wave solution describes the transition of traffic flow from free flow to congestion to free flow.

Through the critical stability conditions in Eq. (12), we get the value of the critical sensitivity $a_{c}$. Based on formulas (27) and (29) in nonlinear analysis, we can obtain the propagation velocity $c$ and the amplitude *A* of the kink–antikink density wave under different transmission delay time $\delta$. The computational values of $a_{c}$,$c$ and *A* corresponding to the CD and OV model for various $\delta$ with $\tau { = 1}$ are listed in Table 1. It can be seen clearly from Table 1 that with the increase of $\delta$, the corresponding values of both $a_{c}$ and $A$ increase gradually in the vehicle-vehicle cooperative driving system. However, in this case, the corresponding propagation velocity value $c$ is constant. This means that in a V2V environment, with the increase in transmission network delay time $\delta$, the possibility of traffic system instability and congestion is increasing.

**Table 1:** The critical sensitivity *a*_*c*_, propagation velocity *c* and the amplitude *A* for various $\delta$ with $\tau { = 1}$.

$\delta$	0	0.1	0.2	0.3	0.4	0.5	0.6	0.7
*a*_*c*_	2.06	2.26	2.47	2.67	2.88	3.08	3.29	3.50
*C*	5	5	5	5	5	5	5	5
*A*	12.50	13.67	14.74	15.74	16.68	17.57	18.41	19.22

## Numerical simulation

Based on the proposed CD and OV model, numerical simulation is carried out under periodic boundary conditions. Therefore, *N* vehicles are set to be evenly divided on the circular road with length *L*, and it is assumed that the first vehicle is disturbed by external small disturbance signals at the initial time^14^:

30 $$x_{1} (0) = 1 \, m,\,\,x_{1} (0) = (n - 1{ )}L/N \, m,{\text{ for}}\,\,n \ne 1$$

31 $$\dot{x}_{n} (0) = V(L/N) \, (n = 1,2, \cdots ,N)$$

The input parameters of the simulation are: $a = 2.1$, $L = 1500$, $N = 100$.

**Figure 2:**
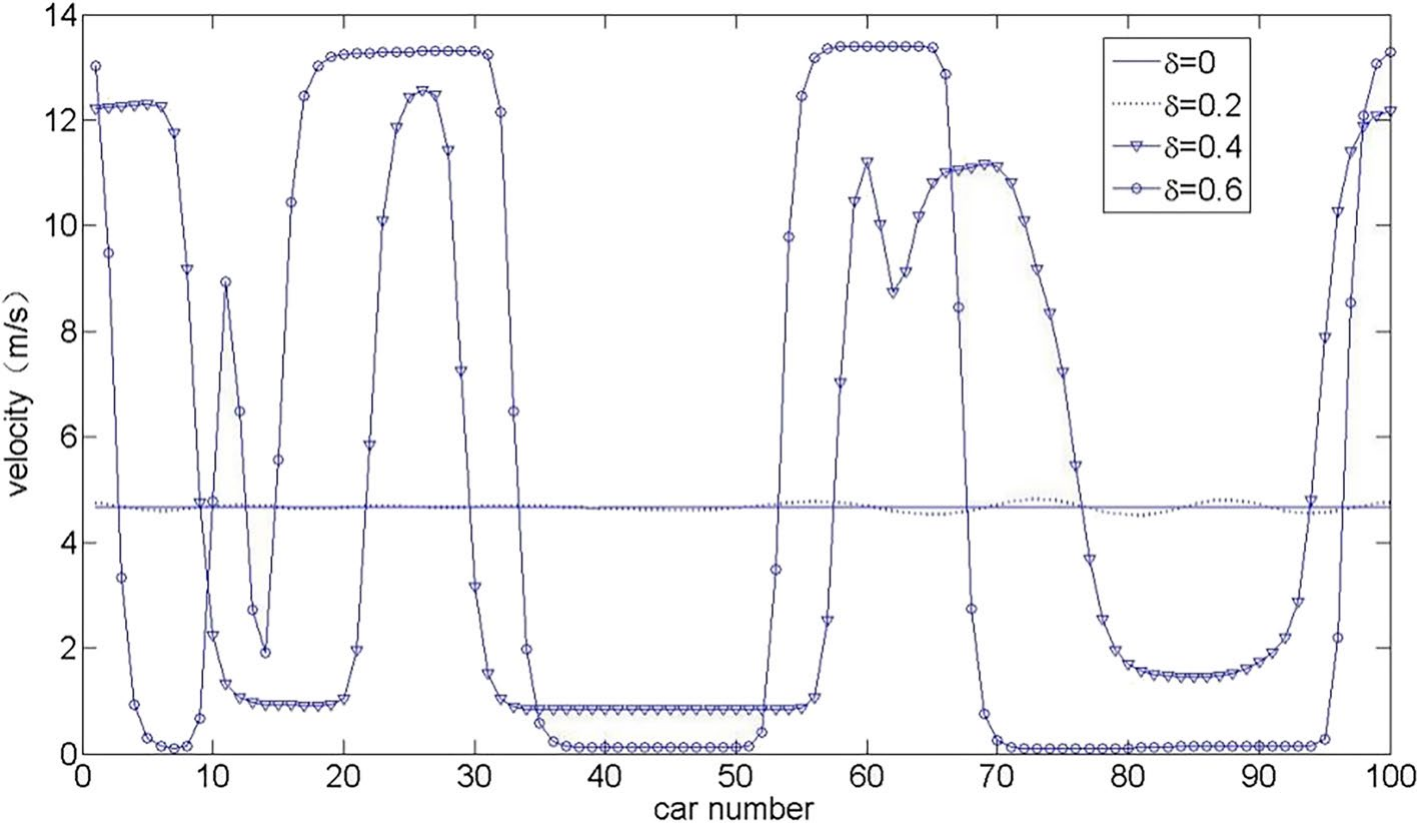
Velocity distribution of all vehicles at a time $t = 600$ s in CD and OV model under different values $\delta$.

**Figure 3:**
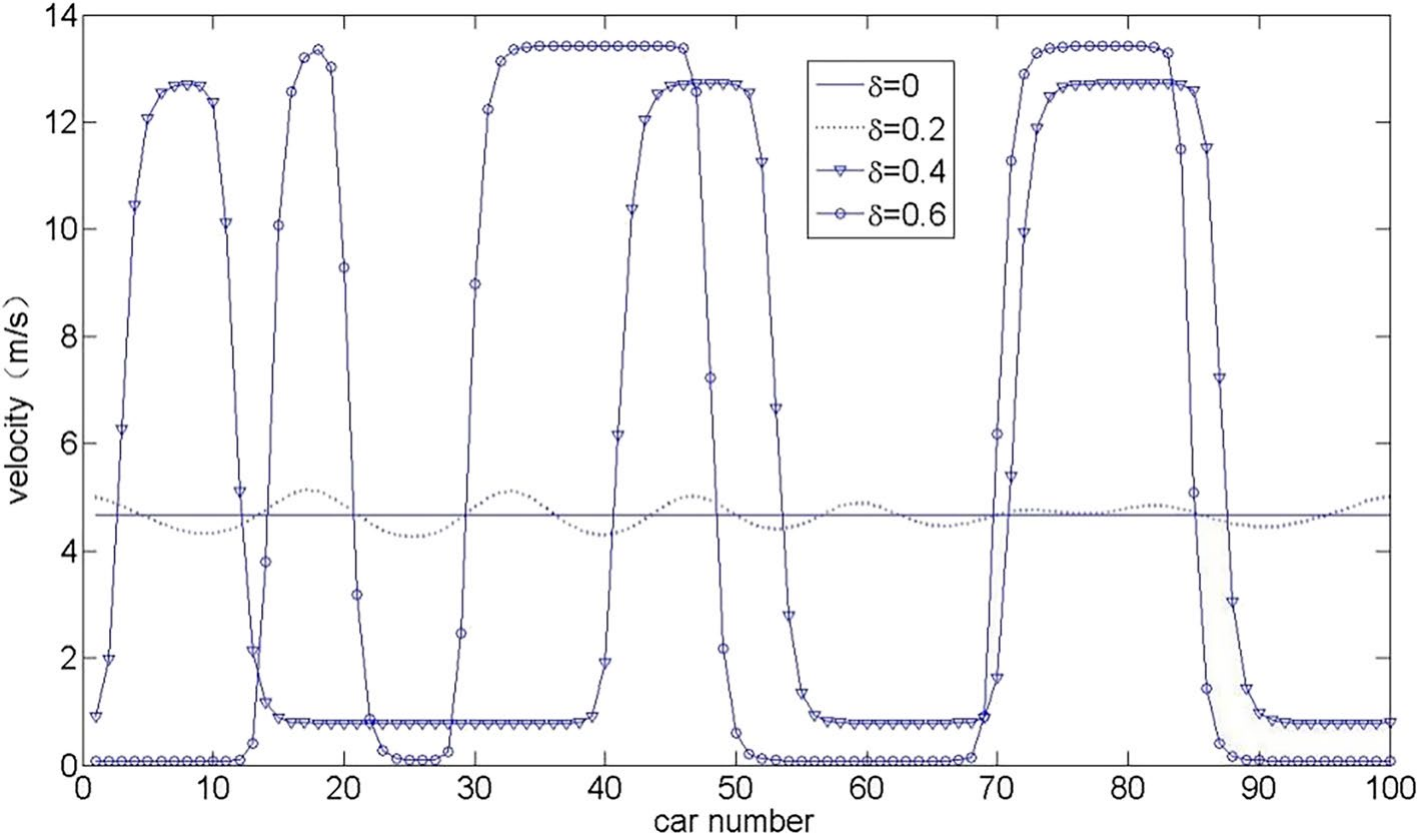
Velocity distribution of all vehicles at a time $t = 1000$ s in CD and OV mode under different values $\delta$.

Figures 2 and 3 show the speed distribution of all vehicles for the CD and OV model corresponding to the time $t = 600$ s and $t = 1000$ s, respectively. When $\delta = 0$, the simulation results were consistent with the OV model^9^. It can be seen from Figs. 2 and 3 that the velocity fluctuation amplitude of the OV model ($\delta = 0$) is smaller than that of the CD and OV model, which shows that the time delay effect caused by transmission network delay has a nonnegligible impact on the cooperation driving process, and the physical motion process of car-following is affected and becomes unstable. At this time, the small disturbance propagates and amplifies in the traffic flow, and forms a kink-antikink wave propagating upstream of the traffic flow with the development of time. In addition, with the increase of end-to-end transmission delay parameter $\delta$, the fluctuation range of the speed of traffic flow gradually increases, and the stability of the traffic flow system deteriorates.

**Figure 4:**
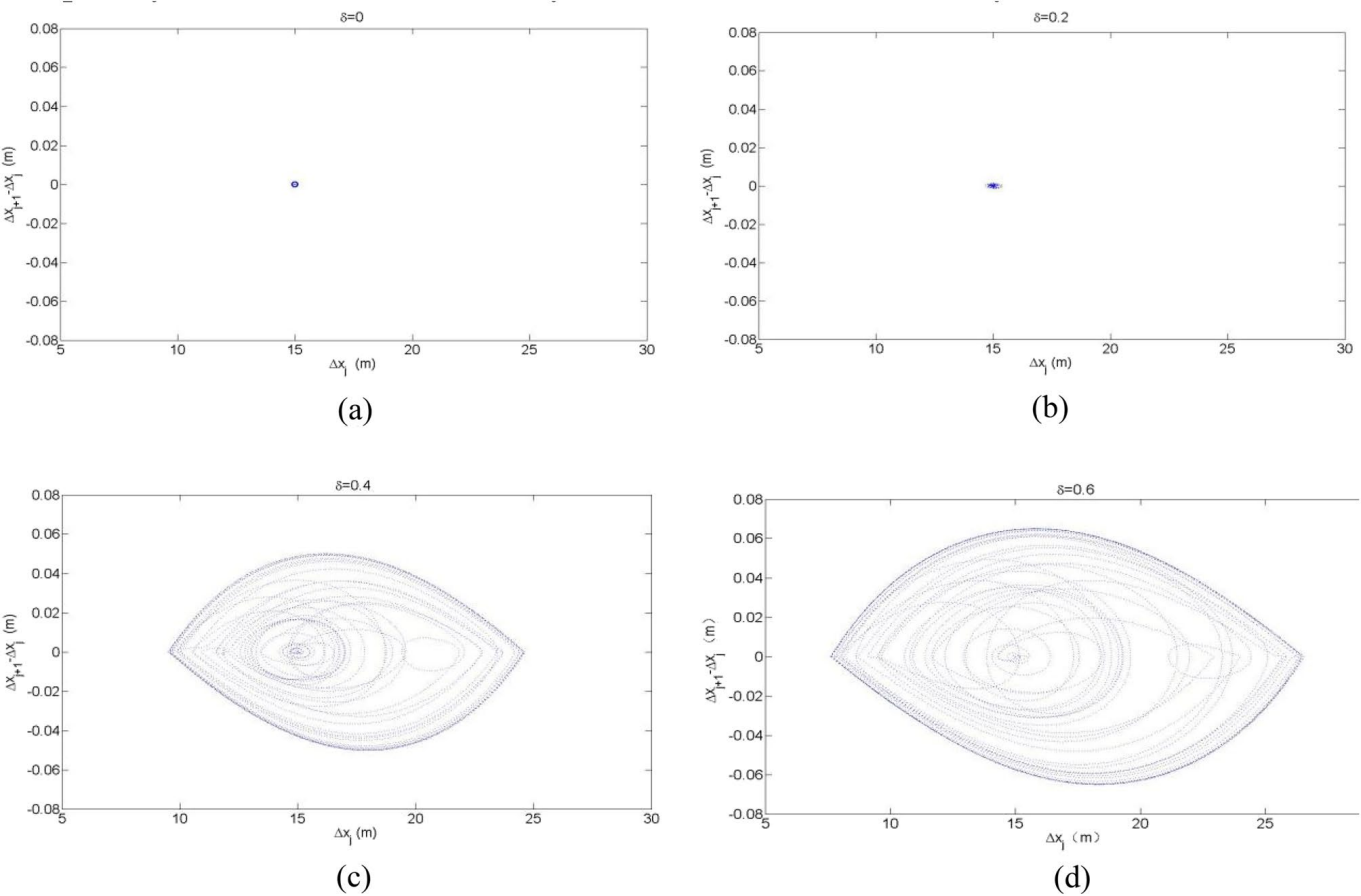
Plot of the headway difference $\Delta x_{j} (t) - x_{j} (t - 1)$ against headway $\Delta x_{j} (t)$ for t = 900–1000 in CD and OV model under different values $\delta$.

Figure 4 shows the trajectory of (($\Delta x_{j} (t) - x_{j} (t - 1),\Delta x_{j} (t)$)) for the CD and OV model when $\delta$ = 0, 0.2, 0.4 and 0.6, respectively. After the system has undergone enough evolution, its spatiotemporal trajectory moves into the closed loop's periodic orbit. Figure 4 illustrates how the closed loop grows larger as the end-to-end transmission delay $\delta$ gradually increases. This indicates that as the vehicle-to-vehicle cooperation driving process progresses, the traffic flow becomes more and more unstable. Therefore, it can be seen that the end-to-end transmission delay of the V2V network has an important impact on the stability of the cooperation driving system. In particular, when $\delta = 0$, corresponding to the completely ideal information transmission situation, the model degenerates into the OV model. Because the model stability condition (13) is satisfied in patterns (a) and (b), the hysteresis loop degenerates into a point, and the traffic flow enters the optimal operation state. This is completely consistent with the analytical result of linear stability.

**Figure 5:**
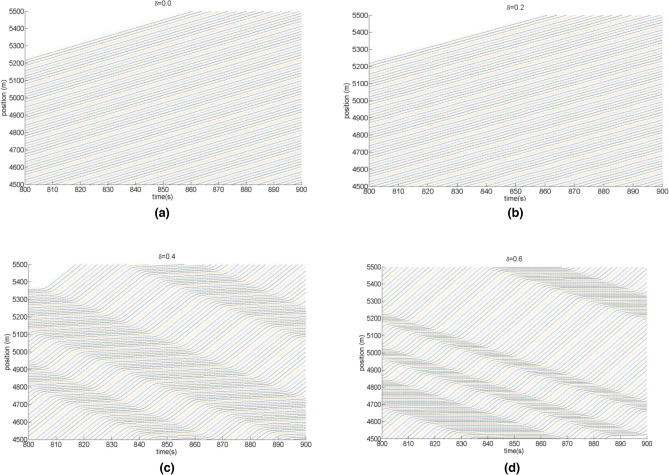
Spatio-temporal evolution of the CD and OV model during the time step of 800–900 corresponding to different end-to-end delays of of communication network, where figures (**a**–**d**) correspond to $\delta$ = 0, 0.2, 0.4 and 0.6, respectively.

Figure 5 shows the spatio-temporal evolution of the CD and OV model corresponding to end-to-end delays $\delta$ in the communication network during the time step of 800–900. From Fig. 5, it can be found that in the ideal case with no end-to-end transmission delay($\delta = 0$), the corresponding model degenerates to the classical OV model^9^, and the subgraph (a) of spatio-temporal evolution shows the traffic flow as equilibrium in the whole space. However, when the end-to-end delay of the network is introduced into the traffic system ($\delta \ne 0$), the anti-interference and stabilizing capability of the traffic system becomes worse, and one can further find from the evolution trend in Fig. 5c,d that as the delay $\delta$ increases, more local clustering appears in the traffic flow field, and the stop-and-go traffic jam distributes more obviously in the whole space. This strongly indicates that the influence of end-to-end communication network delays cannot be neglected in the vehicle-to-vehicle cooperative driving model.

## Conclusion

In the V2V communication environment, the vehicle-to-vehicle cooperative driving process is subject to strict performance constraints on the upper bound of network end-to-end transmission delay. Establishing a vehicle-to-vehicle cooperation model suitable for the end-to-end transmission delay of a V2V network and exploring its mechanism on this basis has strong theoretical significance and practical value. Based on the basic idea of the car following model and considering the time-delay characteristics of the cooperative driving process from information sensing to control decision and then to physical execution, this paper introduces the time-delay parameters representing the end-to-end transmission delay of the network and puts forward the CD and OV model. The stability analysis of the new model is studied, and the stability criterion of the system under the effect of end-to-end transmission delay is obtained. Through the reductive perturbation method, the space–time evolution equation of traffic flow near the critical stable point is obtained, and the density wave solution of the equation is derived, which reveals the propagation and evolution mechanism of traffic congestion caused by the end-to-end transmission delay effect of the network. The numerical simulation verifies the correctness of the stability analysis and nonlinear analysis results. The research shows that the end-to-end transmission delay of the network in the information space has a nonnegligible impact on the stability of the cooperative driving physical process described by the CD and OV model, and the traffic flow stability gradually decreases with the increase of the end-to-end transmission delay of the network, resulting in more and more severe traffic congestion and traffic flow fluctuation. The ability of a time-delay system to suppress small disturbance signals is negatively correlated with the end-to-end transmission delay factor. This fully shows that in the research of vehicle-to-vehicle cooperation driving modeling in a V2V communication environment, the impact effect of network end-to-end transmission delay on system stability needs to be fully considered.

## Data Availability

The datasets/codes are available from the corresponding author upon reasonable request.
